# Intraoperative mapping of pre-central motor cortex and subcortex: a proposal for supplemental cortical and novel subcortical maps to Penfield’s motor homunculus

**DOI:** 10.1007/s00429-021-02274-z

**Published:** 2021-04-19

**Authors:** Prajwal Ghimire, Jose Pedro Lavrador, Asfand Baig Mirza, Noemia Pereira, Hannah Keeble, Marco Borri, Luciano Furlanetti, Christian Brogna, Jozef Jarosz, Richard Gullan, Francesco Vergani, Ranjeev Bhangoo, Keyoumars Ashkan

**Affiliations:** 1 Department of Neurosurgery, King’s College Hospital NHS Foundation Trust, London, UK; 2 Department of Neuroradiology, King’s College Hospital NHS Foundation Trust, London, UK; 3 Neuromonitoring Team, Inomed Neurocare UK Ltd., London, UK

**Keywords:** Motor homunculus, Subcortical map, Craniotomy, Corticospinal tract, Motor cortex

## Abstract

Penfield’s motor homunculus describes a caricaturised yet useful representation of the map of various body parts on the pre-central cortex. We propose a supplemental map of the clinically represented areas of human body in pre-central cortex and a novel subcortical corticospinal tract map. We believe this knowledge is essential for safe surgery in patients with eloquent brain lesions. A single-institution retrospective cohort study of patients who underwent craniotomy for motor eloquent lesions with intraoperative motor neuromonitoring (cortical and subcortical) between 2015 and 2020 was performed. All positive cortical and subcortical stimulation points were taken into account and cartographic maps were produced to demonstrate cortical and subcortical areas of motor representation and their configuration. A literature review in PubMed was performed. One hundred and eighty consecutive patients (58.4% male, 41.6% female) were included in the study with 81.6% asleep and 18.4% awake craniotomies for motor eloquent lesions (gliomas 80.7%, metastases 13.8%) with intraoperative cortical and subcortical motor mapping. Based on the data, we propose a supplemental clinical cortical and a novel subcortical motor map to the original Penfield’s motor homunculus, including demonstration of localisation of intercostal muscles both in the cortex and subcortex which has not been previously described. The supplementary clinical cortical and novel subcortical motor maps of the homunculus presented here have been derived from a large cohort of patients undergoing direct cortical and subcortical brain mapping. The information will have direct relevance for improving the safety and outcome of patients undergoing resection of motor eloquent brain lesions.

**Supplementary Information:**

The online version contains supplementary material available at 10.1007/s00429-021-02274-z.

## Introduction

Homunculus as described by Penfield and Boldrey in 1937 after bipolar direct cortical stimulation (DCS) in 126 awake patients has provided the foundation for intraoperative motor mapping in patients undergoing craniotomy for brain lesions (Penfield and Boldrey 1937). Since then, there has been a range of studies on motor mapping of corticospinal tract (CST) using neuro-physiological, viral tracing, microstructural, cadaveric and intraoperative human and animal studies (Foerster 1936; Farrell et al. 2007; Rathelot and Strick 2009; Desmurget and Sirigu 2015). More recently, there has been further studies using advanced imaging techniques such as deterministic as well as probabilistic tractography to produce a map of the CST, including U fibre connections, highlighting the complex fibre connections and overlap (Berman et al. 2004; Catani 2017). Human connectome project has further provided an advanced interface to characterise the configuration of cortical and subcortical CST (Human Connectome Project 2020). Difference in the size of representation of body-parts within the primary motor cortex has been recognized and is thought to be likely related to each regions’ functional specialisation and fine motor functions (Penfield and Bouldrey 1937; Penfield and Rasmussen 1950; Catani 2017).

Advancements in the DCS and subcortical mapping techniques (monopolar stimulation, dynamic continuous monitoring), allow accurate measurement of the distance from CST during intraoperative stimulation to achieve safe lesion resection and avoid inadvertent injury to CST central to preserving the quality of life of the patients (Seidel et al. 2013; Schuncht et al. 2014; Lavrador et al. 2020). Bello et al. (2008) described subcortical mapping in 57 patients where individual areas of hand, arm and leg were identified during resection of gliomas and accurate identification of CST enhanced surgical performance and safety, maintaining a high rate of functional preservation. Increasingly, a combination of pre-operative [functional magnetic resonance imaging (fMRI), diffusion tensor imaging (DTI), navigated transcranial magnetic stimulation (nTMS)] and intra-operative [neuronavigation, intra-operative ultrasound (ioUS), augmented reality microscope, 5-aminolevulinic acid (5-ALA)] tools are combined with direct intra-operative brain mapping to aid surgical planning for maximal safe resection (Bello et al. 2008; Seidel et al. 2013; Vassal et al. 2013; Schucht et al. 2014; Hervey-Jumper and Berger 2016; Lavrador et al. 2020).

Despite these studies and the data gathered, no updated homunculus nor subcortical map, based on intra-operative direct cortical/subcortical stimulation, has been proposed since the original 1937 work **(**Supplemental Table 1).

In this study, we propose a supplemental motor cortical map and a novel motor subcortical map of the configuration of the corticospinal tract initially described by Penfield (Penfield and Boldrey 1937; Penfield and Rasmussen 1950). These maps, although represent artistic illustrations similar to that presented by Penfield, provide further insight into the cortical and subcortical representation of the body parts, critical for surgery in eloquent brain and for better clinical outcomes. Furthermore, they also form the basis for future large-scale work towards formal probabilistic maps of the cortical and subcortical homunculi.

## Materials and methods

A single-centre retrospective study was performed between January 2015 and January 2020 to collect intraoperative data on consecutive patients who underwent craniotomy for eloquent brain lesions with intraoperative cortical and subcortical motor neuromonitoring at our quaternary referral neurosurgical centre. Patients were consented for intraoperative neuromonitoring including motor mapping. Prior to the surgery, patients underwent a range of pre-operative brain mapping investigations to include fMRI, DTI and nTMS to evaluate the feasibility of lesion resection, determine the best surgical approach and help consent the patient based on individual’s risk profile. At surgery, 3-dimensional (3D) reconstructed structural MRI, tractography from DTI, fMRI hotspots and 3D reconstructed nTMS motor stimulation points were projected onto the brain with augmented reality using ZEISS KINEVO^®^ 900 microscope(CARL ZEISS Meditec AG, Jena, Germany) for the patients. These helped as starting points to define the relationship between the lesions and eloquent motor brain areas, guiding intra-operative mapping with cortical/subcortical stimulation. Thereafter, all positive documented cortical and subcortical points of stimulation confirmed with intraoperative neuromonitoring were recorded and plotted over cartographical maps by utilizing the intraoperative numerically labelled pictures and correlating with intra-operative neuro-navigation MRI, corrected for intra-operative brain shift using the ioUS, and immediate post-operative anatomical T1 post gadolinium MRI. The overlap of stimulated points were also noted. Demographic, clinical and surgical data were collected from patients’ medical records. A PubMed literature review was performed, in order to investigate if a subcortical motor map was previously described, with the MeSH items [(Subcortical stimulation) OR (subcortical mapping) OR (subcortical intraoperative neuromonitoring) AND Motor AND corticospinal tract)] with filters for “articles with abstracts, adult (19 + years), English language articles, human studies and articles published between 1990 and 2020”. The demographic and clinical data were analysed with Microsoft Excel 2020.

### Functional magnetic resonance imaging (fMRI)

Three different tasks were performed: lip smacking, finger-tapping and foot rocking (Supplemental Figure 1). The cortical activated areas were used to constrain the probabilistic tractography.

### Diffusion tensor imaging (DTI) and deterministic tractography

DTI sequences were obtained to reconstruct CST. The parameters utilised for obtaining diffusion tensor imaging were *b*-value: 1500; diffusion directions: 64; diffusion mode; multi-directional diffusion weighting; field of view: 32 cm; voxel size, 2.5 × 2.5 × 2.5 mm; TR/TE:9500/86; scan time:11:35 min. The deterministic tractography was modelled in 3D with StealthViz Medtronic Software (Minneapolis, Minnesota, USA) (Lavrador et al. 2020). The dissection of the corticospinal tract was performed according to the regions of interest (ROIs) between the motor cortex and the ipsilateral half of the medulla oblongata below the middle cerebellar peduncle (Supplemental Figure 6). Constrained probabilistic tractography for complex motor function and cortical/subcortical CST was performed with MRTrix3 opensource Software (Tournier et al. 2019) (Supplemental Figure 2).

### Navigated transcranial magnetic stimulation (nTMS)

Preoperative nTMS was utilised for pre-operative motor mapping, using Nexstim TMS, v.4.3.1 (Nexstim, Helsinki, Finland) (Jung et al. 2019). nTMS was obtained with single-pulse sequence delivered using a figure-of-eight coil, and the motor mapping was performed at 105% of the determined resting motor threshold (Jung et al. 2019). The nTMS preoperative positive motor responses were transformed in 3D objects (StealthViz Medtronic software, Minneapolis, Minnesota, USA) superimposed to the 3D tractography model of the CST on the Medtronic Stealth Station S7/S8 neuronavigation software/machine (Minneapolis, Minnesota, USA) (Supplemental Figure 3).

### Cortical mapping

**Fig. 1:**
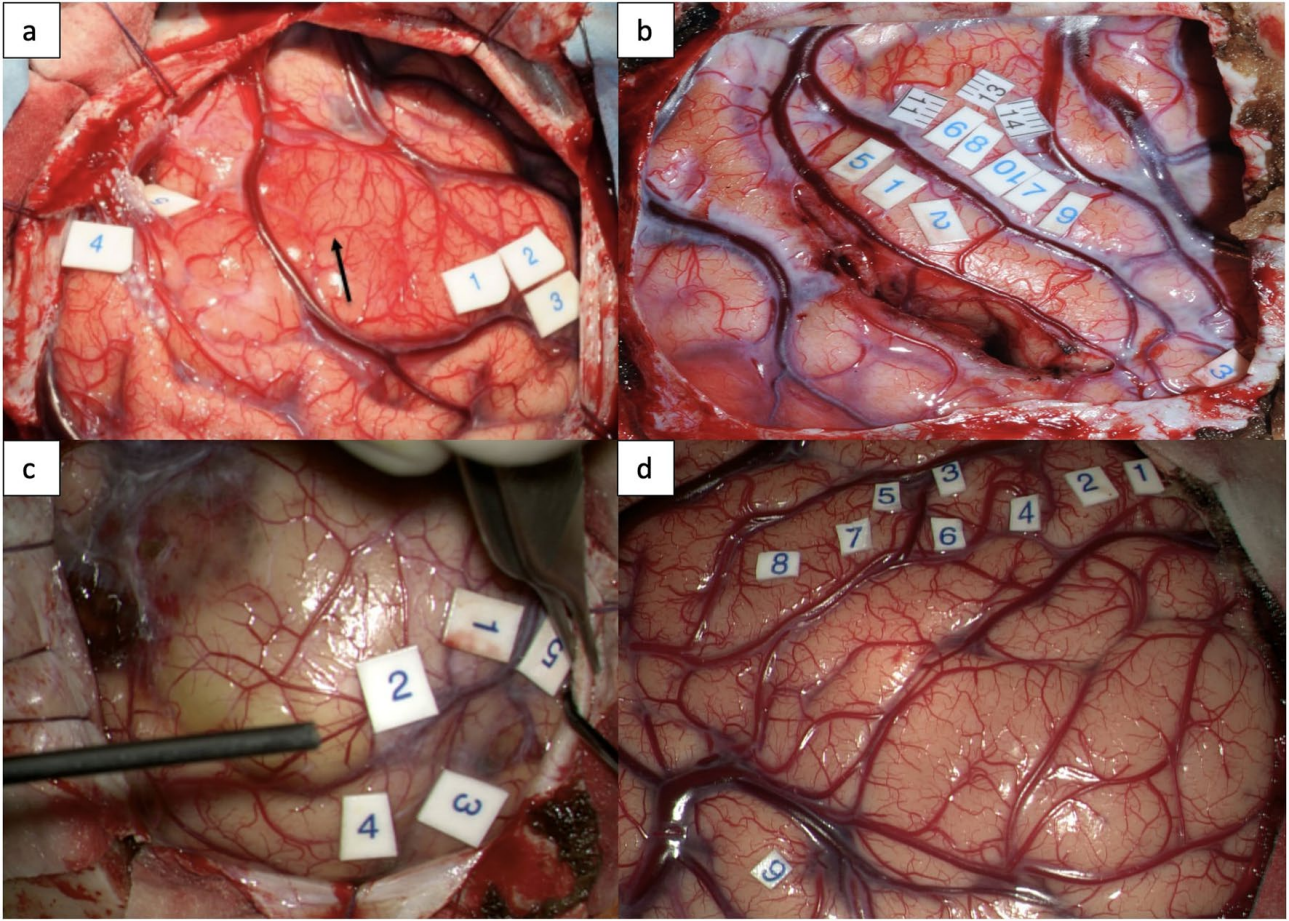
Cortical stimulation with monopolar probe stimulating different areas of motor cortex **a** 1,2,3: hand and forearm 4,5: foot and leg **b** 6,7,8,9,10,11,13,14- hand knob (ADM, FDI, APB, forearm, deltoid) 1,2,5- post central gyrus **c **1,2: intercostal muscles 3,4: deltoid 5: foot **d** 1,2: arm, forearm, hand 3,4,5: hand, face 6,7: face

Cortical motor evoked potentials (cMEPs) were recorded with direct electrical monopolar stimulations of primary motor cortex (Fig. 1). Train of five pulses, positive pulse form, inter-stimuli interval of 4.0 ms, pulse width of 0.5 ms, 1 Hz and anodal pole were the parameters for stimulations. Continuous cMEPs were monitored using a four-contact strip electrode positioned over the motor cortex recording stable MEPs at the motor threshold. Muscles monitored during intraoperative neuromonitoring were orbicularis oris, masseter, tongue, cricothyroid, deltoid, brachioradialis/flexor carpi ulnaris (BR/FCU), abductor pollicis brevis/abductor digiti minimi (APB/ADM), first dorsal interosseous (FDI), intercostals, quadriceps femoris, tibialis anterior, abductor hallucis.

### Subcortical mapping

**Fig. 2:**
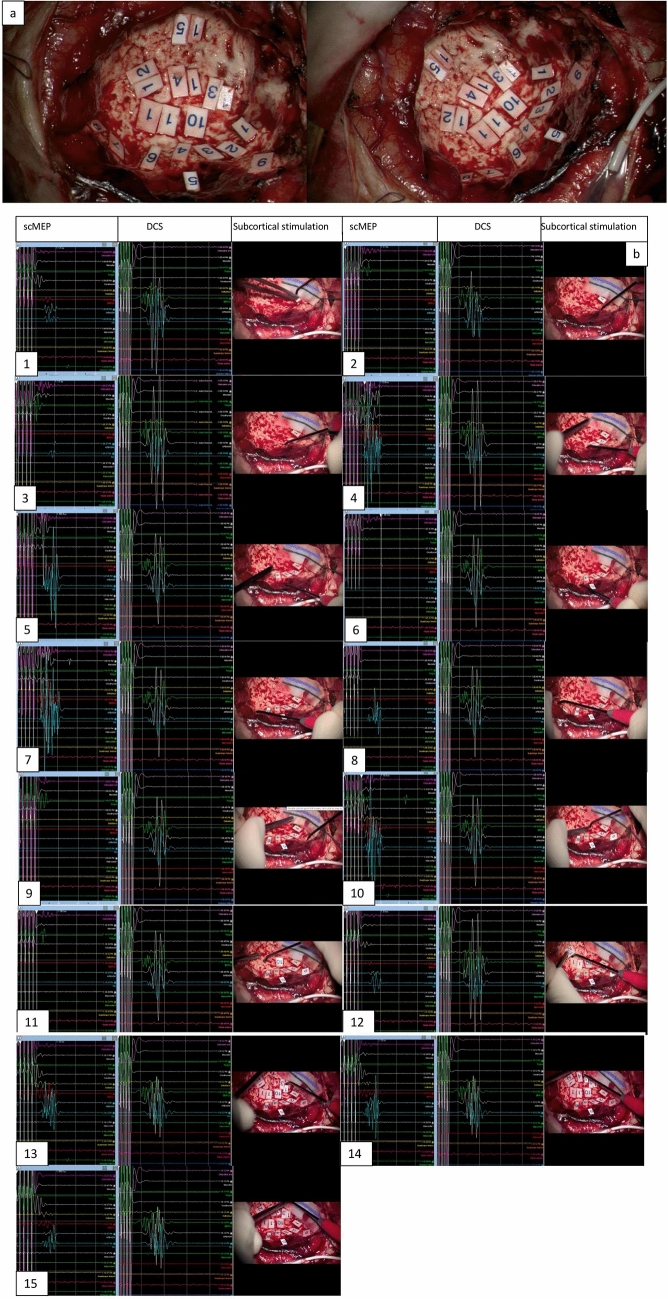
**a** Subcortical stimulation in the resection cavity demonstrating subcortical mapping of the corticospinal tract with contact numbers (1–15); Strip electrode over the primary motor cortex*.* **b** Corresponding mapping/stimulation positive areas (1–15) of intraoperative MEPs for different parts of the subcortical homunculus along with their corresponding motor thresholds*; *Subcortical MEP recording: *X* axis: time (ms), *Y* axis: amplitude (µV); Strip Direct cortical Stimulation (DCS) recording: *X* axis: time (ms), *Y* axis: amplitude (µV); 1—upper limb (8 mA), face (5 mA); 2—upper limb (8 mA), face (6 mA); 3—upper limb (7 mA), face (2 mA); 4—upper limb (5 mA), face (2 mA); 5—upper limb (7 mA), face (4 mA); 6—face (2 mA); 7—upper limb (6 mA), face (5 mA); 8—upper limb (8 mA); 9—face (6 mA); 10—upper limb (7 mA), lower limb (10 mA), face (6 mA); 11—face (8 mA); 12—upper limb, face (13 mA); 13—upper limb (8 mA), face (7 mA); 14—upper limb (12 mA), face (11 mA); 15—upper limb (19 mA), face (17 mA)

Subcortical motor evoked potentials (scMEPs) were recorded using modified monopolar suction probe and monopolar probe (Parameters were same as cMEP recording with cathodal pole) (Seidel et al. 2013; Schucht et al. 2017; Lavrador et al. 2020) (Fig. 2). Muscles monitored were same as on cortical mapping.

### Intraoperative ultrasound

Ultrasound images were obtained intraoperatively prior to corticotomy and at the end of resection with Esaote Ultrasound Machine (Esaote, Genova, Italy) to delineate the margins of the tumour and post resection cavity. It provided the configuration, depth and location of the lesion with respect to the motor cortex and subcortical CST identified with subcortical stimulation and helped to correct for any intra-operative brain shift (Supplemental Figure 4).

## Results

**Fig. 3:**
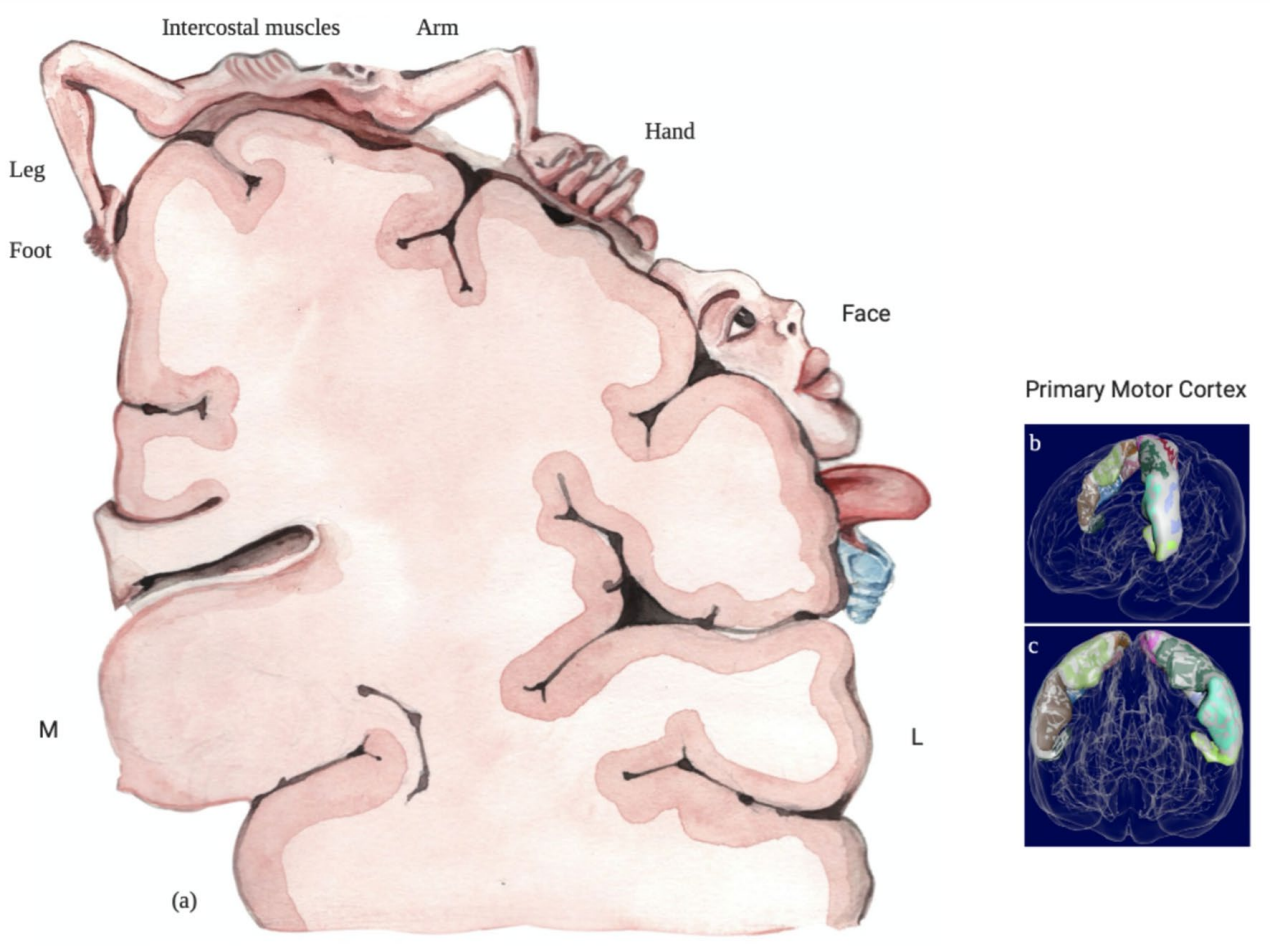
Proposed supplemental cortical motor homunculus including the cortical somatotopy of intercostal muscles: **a** cortical motor representation. **b, c** Primary motor cortex reconstructed with Meshlab opensource software (Cignoni et al. 2008) with the data available from The Human Brainnectome Atlas (Fan et al. 2016)

**Fig. 4:**
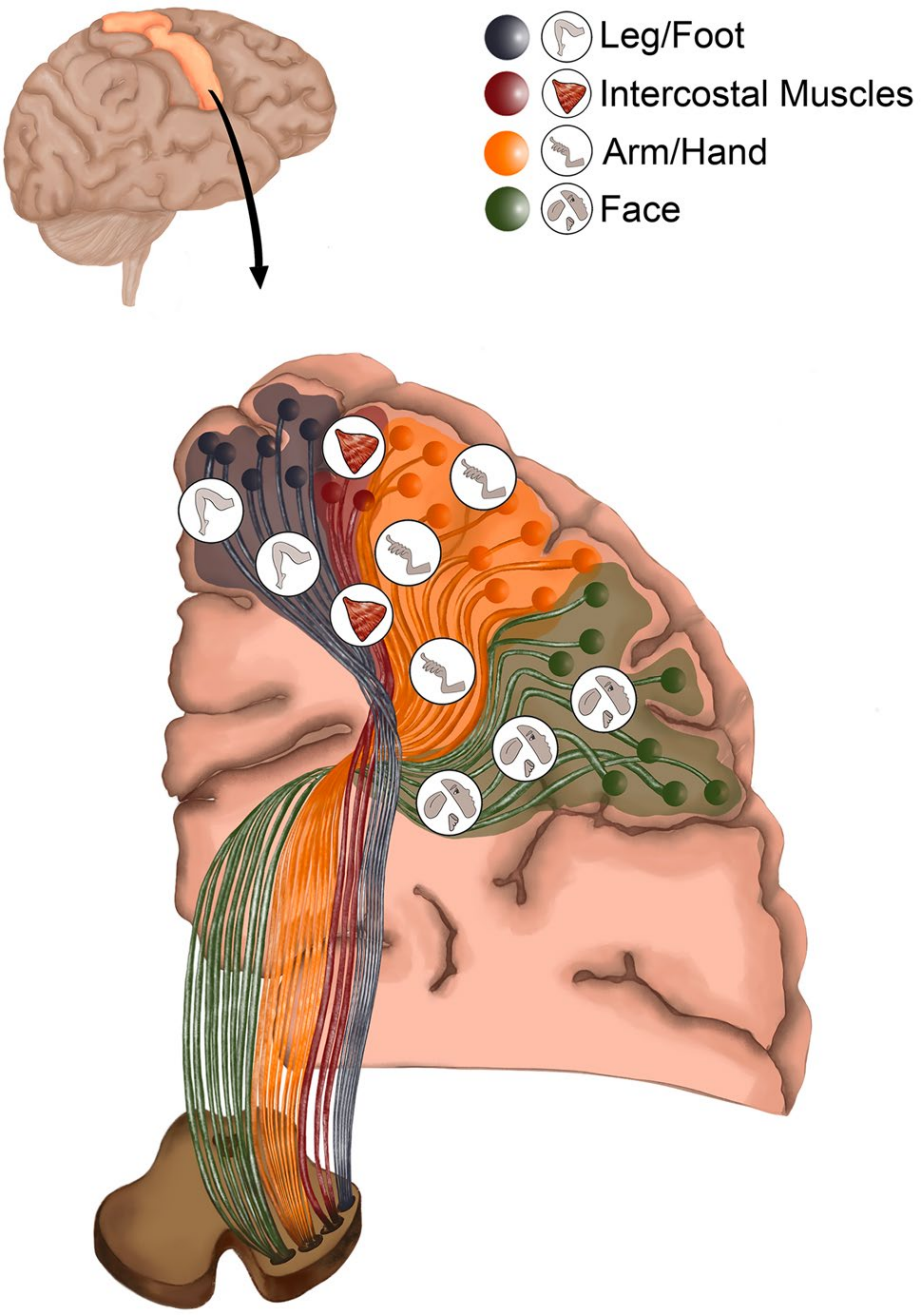
Illustration demonstrating intraoperative subcortical Corticospinal Tract (isCST) (the novel “Clinical subcortical motor homunculus”)

Table 1 summarises the demographics of the 180 consecutive patients studied. All patients underwent pre-operative T1 pre- and post-gadolinium, T2 and FLAIR imaging. 45 patients had DTI, 5 patients had complex motor task fMRI and 39 patients had nTMS. The motor threshold during stimulation represented the estimated distance from the clinically detected anatomical bundle of fibres of CST (1 mA = 1 mm). Cortical motor threshold of DCT of motor cortex/CST was recorded as a mean of 7.2 mA (range 0.8–25 mA, *n* = 180). Cortical and subcortical mean stimulation threshold for leg (c8.4 mA, range 2–17 mA, *n* = 14; sc5.8 mA, range 3–13, *n* = 11), foot (c7 mA, range 5–12 mA, *n* = 17; sc6.5 mA, range 2–14 mA, *n* = 12), intercostal muscles (c10 mA, range 6–14 mA, *n* = 6; sc6 mA, range 4–12 mA, *n* = 7), arm (c7.6 mA, range 0.8–13 mA, *n* = 21; sc4.3 mA, range 2–6 mA, *n* = 6), hand (c8.7 mA, range 0.8-20 mA, *n* = 47; sc6.6 mA, range 5–8.5 mA, *n* = 20) and face (c7.4 mA, range 2–11 mA, *n* = 24; sc5.7 mA, range 0.5–8, *n* = 12) were recorded during the procedures (Tables 2, 3). The stimulated cortical and subcortical points were then plotted in the diagrammatic representation of cortex and subcortex with individual areas stimulated. The points were then assembled into cartographic maps to demonstrate a clinical cortical and subcortical map of intraoperative corticospinal tract (Figs. 3, 4).

**Table 1:** Demographics of the patient data

Demographics	Data
Mean age (years) (with range)	50 (16–79)
Laterality of the lesion	*N* (%)
Right	97 (53.8%)
Left	83 (46.2%)
Gender	*N* (%)
Male	105 (58.4%)
Female	75 (41.6%)
Pathology of the lesion	
Glioma	
High Grade (III, IV)	104 (57.7%)
Low Grade (I, II)	41 (23%)
Meningioma	8 (4.4%)
Metastasis	25 (13.8%)
Vascular malformations	2 (1.1%)

**Table 2:**
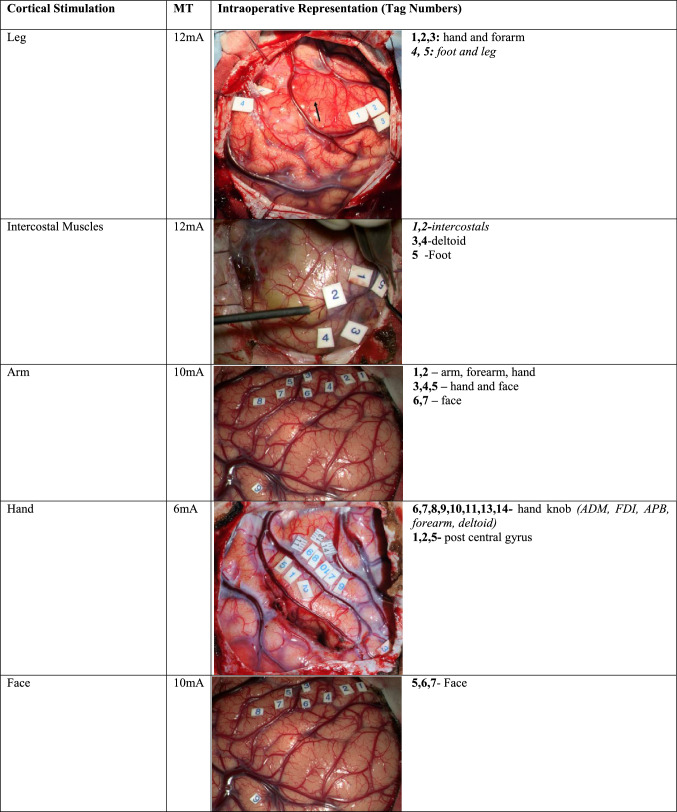
Cortical stimulation of motor cortex areas with their thresholds

*Black arrow* tumour causing expansion of motor/pre-motor cortex, *ADM* abductor digiti minimi, *FDI* first dorsal interossei, *APB* abductor pollicis brevis, *MT* motor threshold

**Table 3:**
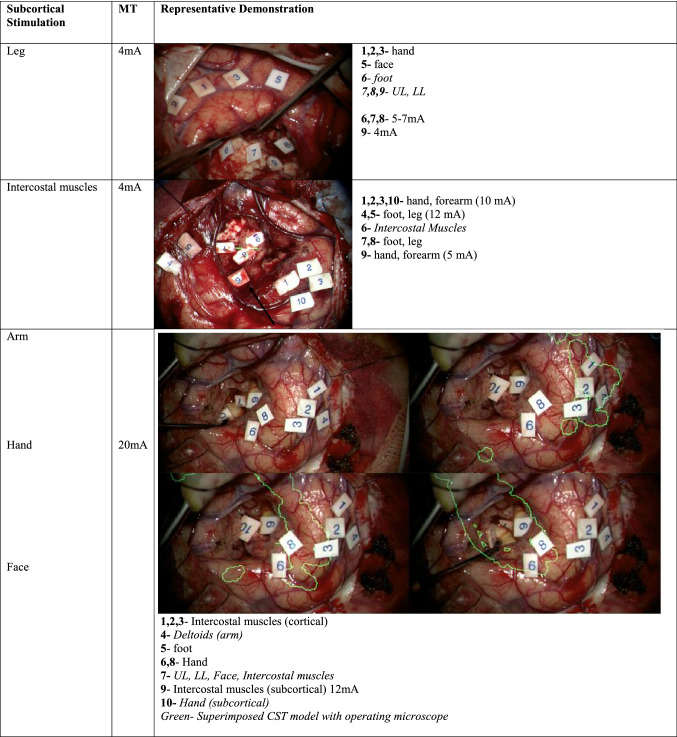
Subcortical stimulation of motor subcortex areas with their thresholds

*UL *upper limbs,* LL *lower limbs,* MT *motor threshold,* CST *corticospinal tract

### Cortical and subcortical stimulation of intercostal muscles

As a contribution to the previously described homunculus, we present, for the first time in the literature, a detailed representation, at both cortical and subcortical level, of the intercostal muscles demonstrated during routine neurophysiological mapping and monitoring (Supplemental Table 2). An example of isolated subcortical stimulation recording (scMEP) during intraoperative subcortical mapping with electrode placed in the intercostal muscles is shown in Supplemental Figure 5. These findings aided in the illustration of the proposed maps.

### Literature review

PubMed literature review resulted in identification of 141 articles. The articles were reviewed to identify data on subcortical mapping of specific motor areas (hand, arm, leg, trunk, face) of patients who underwent craniotomy for brain lesions. Articles that did not show individual-motor-area (hand, arm, leg, trunk, face) subcortical mapping were excluded. Six articles were identified and have been summarised in Supplemental Table 1. There was no literature on a map for the subcortical motor areas.

### Proposed homunculus maps

#### Proposal of supplemental map of cortical motor region (clinical cortical motor homunculus)

The proposed supplemental cortical map consists of new addition of cortical representation of intercostal muscles in the existing Penfield’s motor homunculus (Fig. 3).

#### Proposal of map of intraoperative subcortical corticospinal tract (isCST) (clinical subcortical motor homunculus)

The proposed subcortical novel map of the corticospinal tract along the corona radiata (Fig. 4) provides an insight into the configuration of CST fibres and highlights the significant differences between the cortical orientation and subcortical orientation of the CST fibres.

## Discussion

### Motor pathway topography: current knowledge

Understanding the topography of the motor pathway at both cortical and subcortical levels is crucial for neurosurgeons during surgery for motor eloquent brain lesions if risk of deficits is to be minimised. Penfield’s 1937 description of the arrangement of the motor function onto the homunculus, although a simplified map, provides a representative image of the motor arrangement of function onto the precentral cortex (Penfield and Boldrey 1937). Since then, human clinical studies and cadaver anatomical studies have provided further insight into the complexity of the motor cortex and overlap of function (Farrell et al. 2007; Catani 2017). There has been recognition of dynamic, rather than the traditional static, representation of the body parts on the motor cortex, especially for complex motor movement (Perez and Rothwell 2015; Schellekens et al. 2018) as well as significant overlapping of motor representation in the supplementary motor area (SMA), premotor, primary motor and parietal cortex (Cunningham et al. 2013). Furthermore, contrary to the traditional orientation of body parts on motor cortex, multiple directions of the representation have now been described: lateral-anterior-ventral direction along the precentral gyrus and central sulcus, reflecting a shift from lower- to upper-body muscles (Verstynen and Sabes 2011). Catani recently described a detailed reappraisal of the original Penfield’s findings, suggesting overlaps between the trasitional areas and cross over to the somatosensory cortex along with description of role of U fibres (Catani 2017). Non-human primate studies, using viral tracers, have added additional dimensions to this complexity by defining phylogenetically new M1 and old M1 cortex (Rathelot and Strick 2009). Despite the spectrum of the new knowledge and techniques, from the practical point of view, the original Penfield’s motor homunculus remains central as a starting point when planning surgery for lesions in and around motor cortex, and DCS continues as the gold standard for intra-operative refinement. Thus, Duffau and colleagues, through DCS studies, were recently even able to demonstrate the concept of negative motor response whereby movement arrest was induced at specific stimulation points in the pre-central gyrus (Rech et al. 2019).

### Subcortical corticospinal tract (sCST): literature thus far

Unlike the motor cortex, our understanding of the detailed functional anatomy of the white matter motor tracts is at an earlier stage. Cadaveric anatomical and imaging studies have shown that corticospinal tract (CST) predominantly begins in the pre-central gyrus (M1), making its way caudally through the corona radiata into the internal capsule, condensing the fibres with a unique formation and thus producing a swirl-like configuration with the twist of the fibres subcortically (Ebeling and Reulen 1992; Kim and Pope 2005; Yamaha et al. 2007; Verstynen et al. 2011; Zolal et al. 2012; Chenot et al. 2019).The CST undergoes a rotation whereby the most medially located fibres at the cortical level (topographically—lower limb) become posterior at the level of the internal capsule and lateral at the level of the cerebral peduncle (Englander et al. 1975; Ebeling and Reulen 1992; Catani and Thiebaut 2008; Chenot et al. 2019). Primate studies have demonstrated the complexity of organisation of the CST fibres corresponding to the functional body areas and its dynamic nature (Graziano et al. 2002). Understanding the detailed anatomy of these tracts is, therefore, essential for safe surgery for lesions in or around the subcortical motor pathways. In the recent years, DTI has proved useful as a starting point to understand the relationship between the CST and the lesions with DTI based maps of subcortex emerging as useful tools for pre-operative planning (Berman et al. 2004; Okada et al. 2006; Rosenstock et al. 2017; Oda et al. 2018; Chenot et al. 2019). The accuracy of the image-based technology, however, remains less than what is required for confident safe surgery and intra-operative direct stimulation of subcortical motor pathways remain the gold standard.

### Current contribution

Our supplementary cortical motor homunculus presented here, provides a useful addition to the original Penfield’s homunculus by clearly demonstrating the motor cortical area for the intercostal muscles, previously not described in detail. We first reported mapping of intercostal muscles as a technical note (Ghimire et al. 2019). This has now been replicated and mapped in detail in this paper (Supplemental Table 2). Understanding the cortical topography of intercostal muscles is important to avoid inadvertent damage during the surgery which could lead to paralysis of respiratory muscles and the well-recognised post-operative respiratory complications (Ghimire et al. 2019). Despite the limited number of papers published on subcortical stimulation (Supplemental Table 1), accurate intraoperative identification of cortical and subcortical boundaries has been challenging with no clear subcortical map, along the same line as the cortical motor homunculus, has been described. We therefore aimed to address this using the data from our large cohort of patients as demonstrated in our novel subcortical map. In this illustration and based on our intra-operative direct stimulation of CST, the motor areas of leg shifted from medial to the posterior, motor areas of hand/forearm shifted from superior to the centre of the condensed bundle and the face representation shifted from lateral to the anterior aspect of the condensed bundle demonstrating the change in CST configuration during the descent of fibres within the corona radiata. There were further changes in configuration prior to reaching the internal capsule: the leg area shifted from posterior to the midline position with the face area shifting anteriorly and the arm area shifting posteriorly as progressing into the known configuration of fibres in the genu and posterior limb of internal capsule. The subcortical map thus generated, we hope will aid in a better understanding of the functional anatomy of the CST and help safe surgery for motor eloquent lesions.

As our understanding of the biology of brain lesions, particularly gliomas, evolve and concepts such as survivorship and preservation of quality of life, quite rightly, gain centre-stage, there is an ever-increasing need for maximal safe resection. This remains a challenge for lesions in and around the motor pathways. Understanding the cortical and subcortical functional anatomy is therefore crucial and it is best derived from intra-operative direct stimulation of motor pathways. Our findings from a large cohort of patients presented as an updated motor homunculus and a novel subcortical map will aid neurosurgeons in their quest to achieve the best outcome possible for these patients.

### Limitation of the study

The proposed maps here are artistic illustrations of motor pathways, based on intra-operative stimulation similar to Penfield’s original homunculus, which we hope will further guide neurosurgeons during eloquent cortical and subcortical brain tumour surgery. The ultimate goal, however, remains the creation of a formal probabilistic atlas of the cortical and subcortical homunculi. Large scale, prospective studies with normalisation of stimulation sites into a template space are required to achieve this. We hope the work presented here will help generate interest in such future endeavours.

## Conclusion

Penfield’s motor homunculus represented a landmark in understating the functional anatomy of the motor cortex with real implications for surgery in motor eloquent brain. After an almost a century, however, there is now time for a reappraisal. Our data from a large cohort of patients undergoing modern intra-operative stimulation of motor pathways, the widely accepted standard methodology for brain mapping based on accuracy and clinical relevance, generated a supplementary updated motor homunculus and a novel subcortical motor map. Our finding will aid surgical planning for lesions in or around the motor pathways, to reduce the risks and increase the extent of resection. Further prospective multicentre studies are required to validate the map towards routine utilisation in clinical practice and generation of probabilistic atlases.

## Supplementary Information

Below is the link to the electronic supplementary material.

Supplementary file1 (DOCX 12271 kb)
