# Supplementary material for: Intraoperative mapping of pre-central motor cortex and subcortex: a proposal for supplemental cortical and novel subcortical maps to Penfield’s motor homunculus
Source: Brain Struct Funct. 2021 Apr 19;226(5):1601–11. doi: 10.1007/s00429-021-02274-z (PMC8096772; doi:10.1007/s00429-021-02274-z)
Supplement: Supplementary file 1 — Supplementary file1 (DOCX 12271 kb) [file 429_2021_2274_MOESM1_ESM.docx]

**
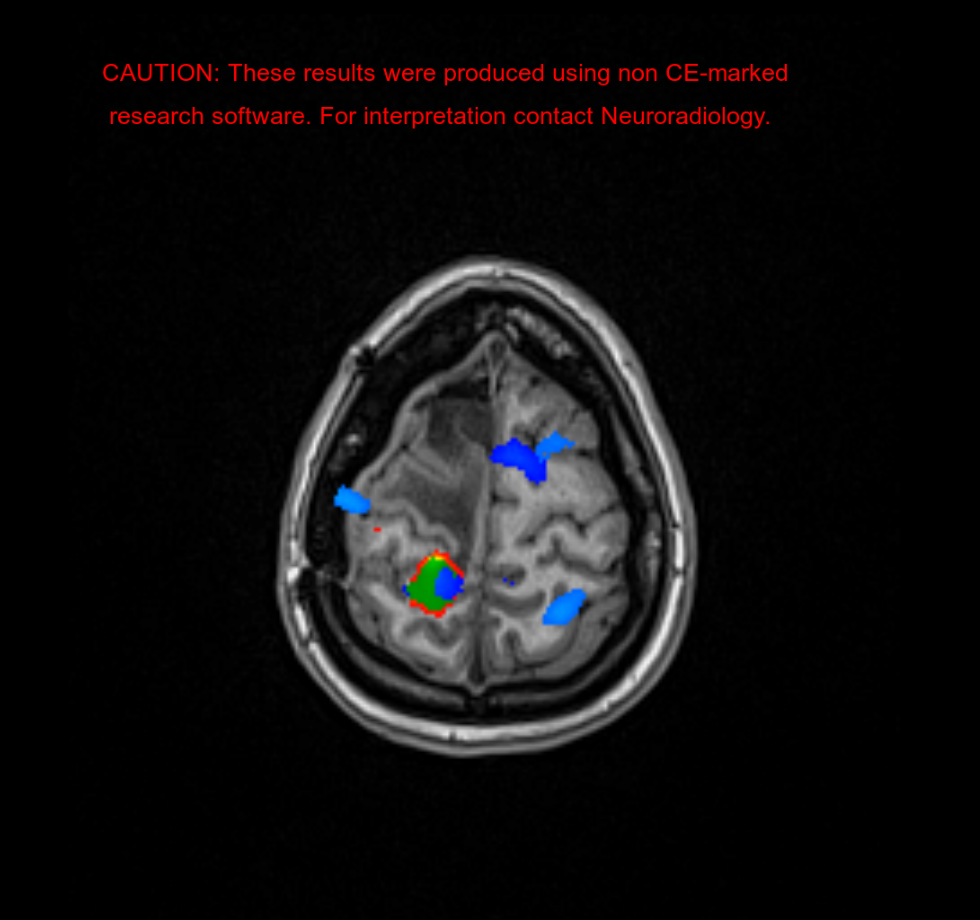

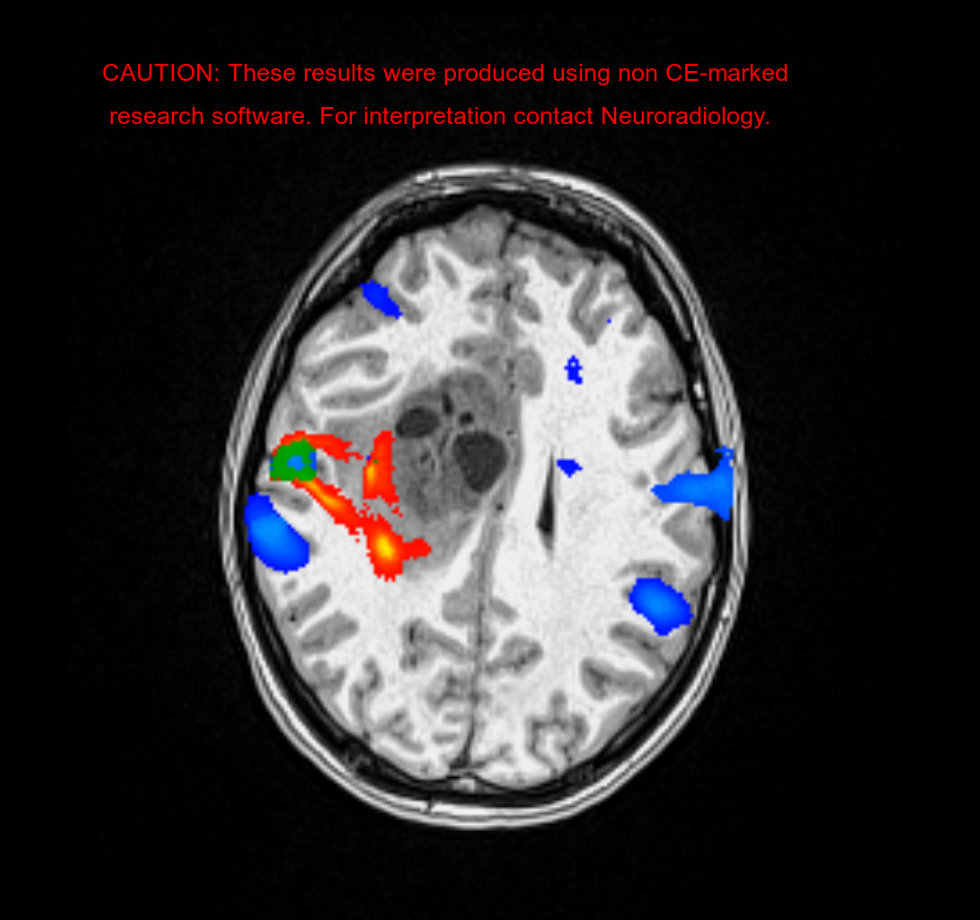

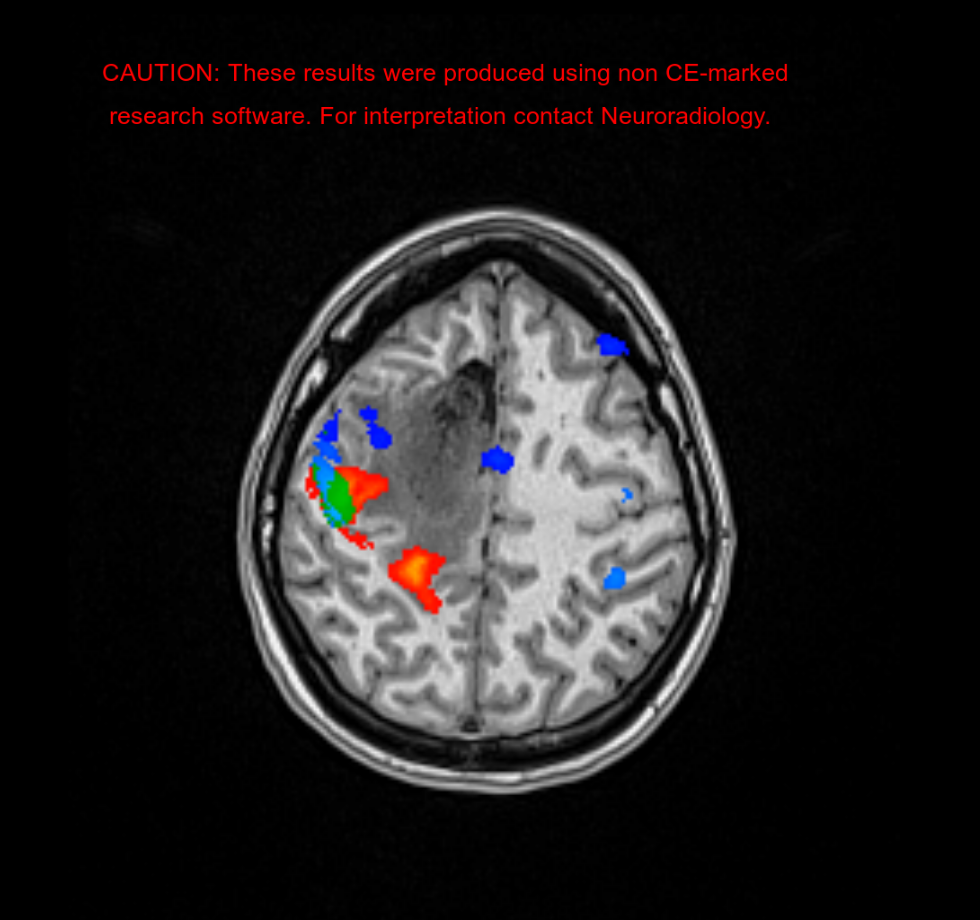
Supplemental figure 1 (a,b,c): fMRI demonstrating complex motor function mapping (a-foot rock task, b-Finger tapping, c-lip smacking) demonstrating cortical complex motor function interacting with CST (*coloured areas correspond to activation clusters as per fMRI analysis for each motor activity*) *(****36y left-handed female with recurrent right frontal transformed WHO Grade III IDH 1 mutant MGMT methylated anaplastic gemistocytic astrocytoma who underwent subtotal resection*)

c

b

a

**
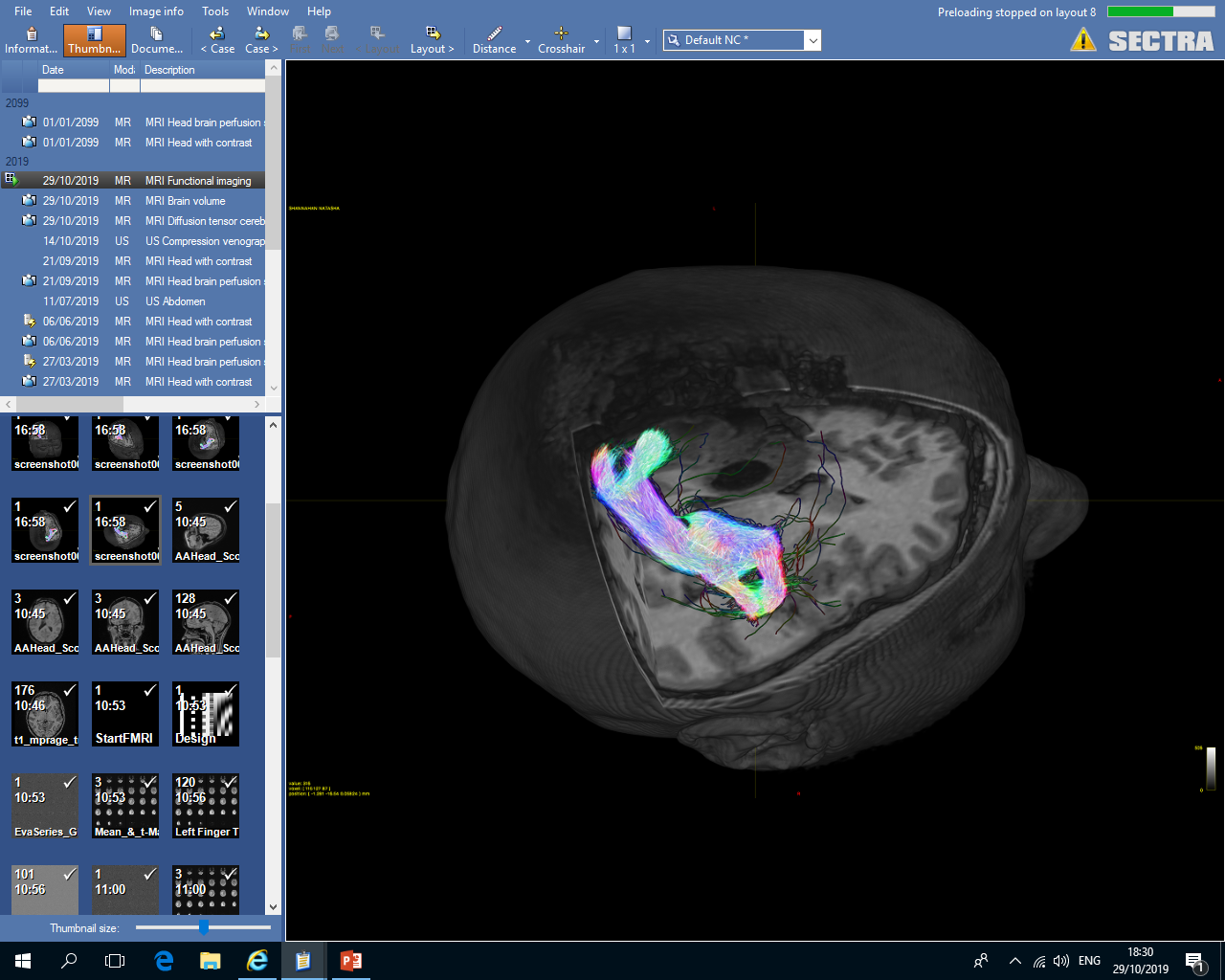

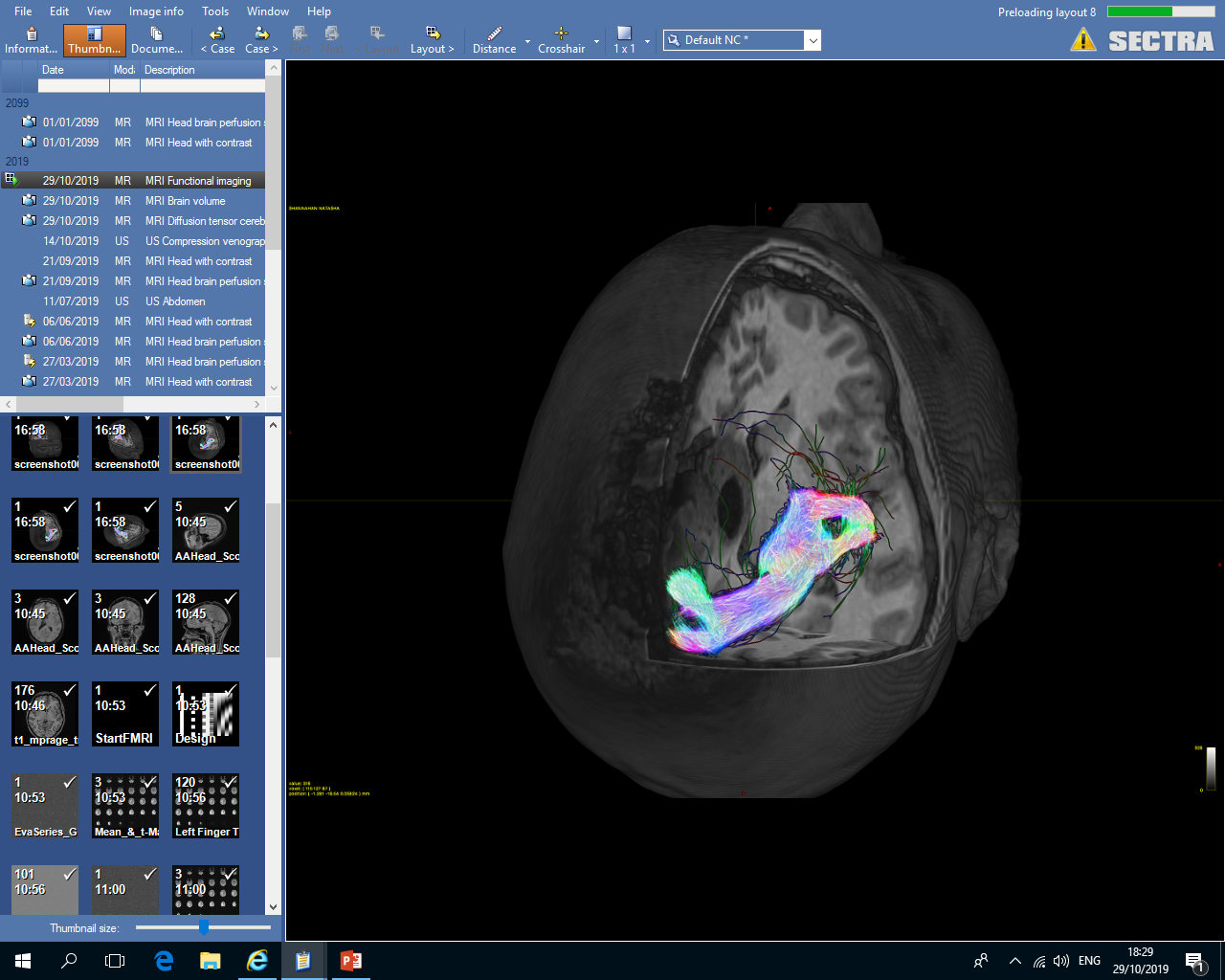

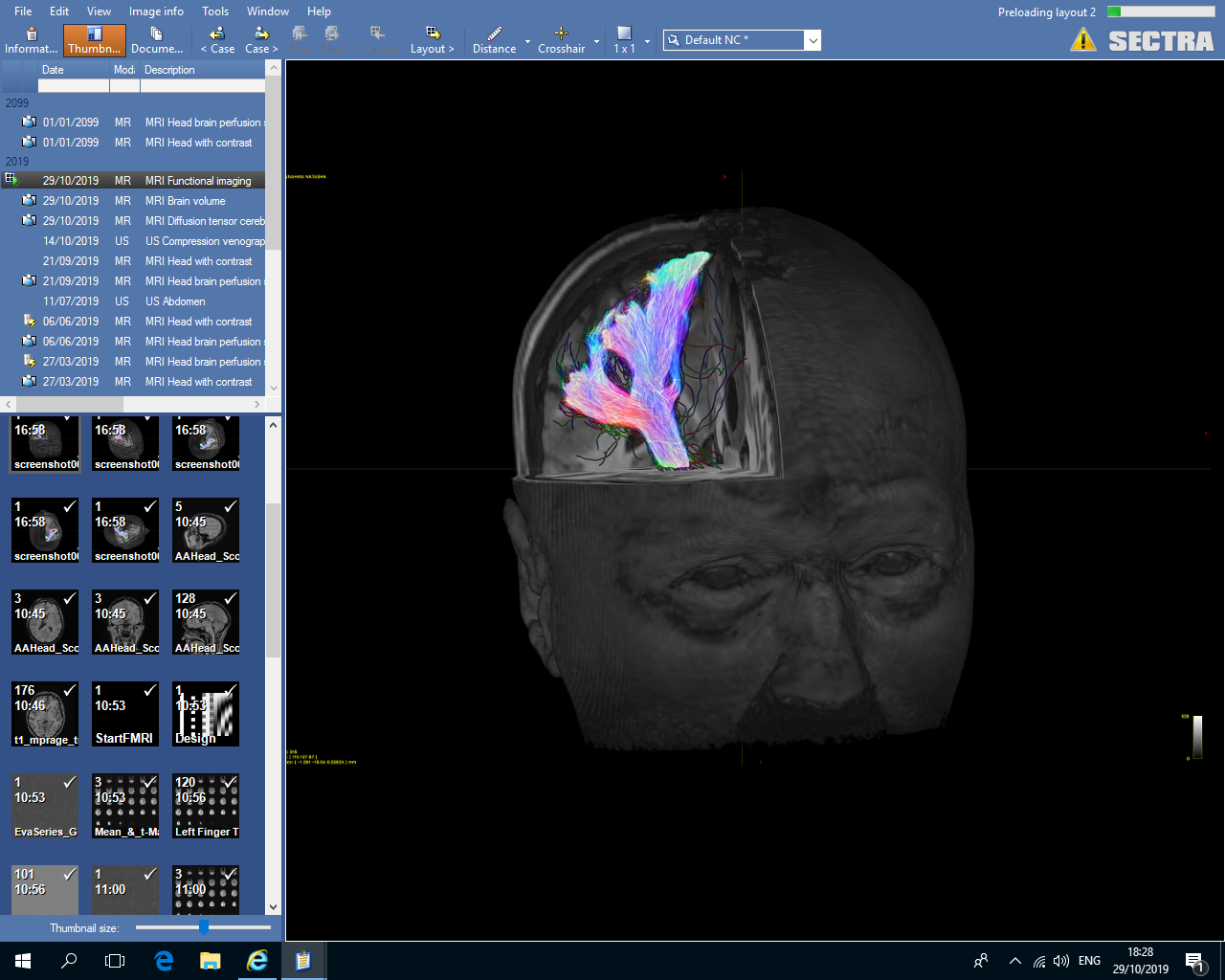

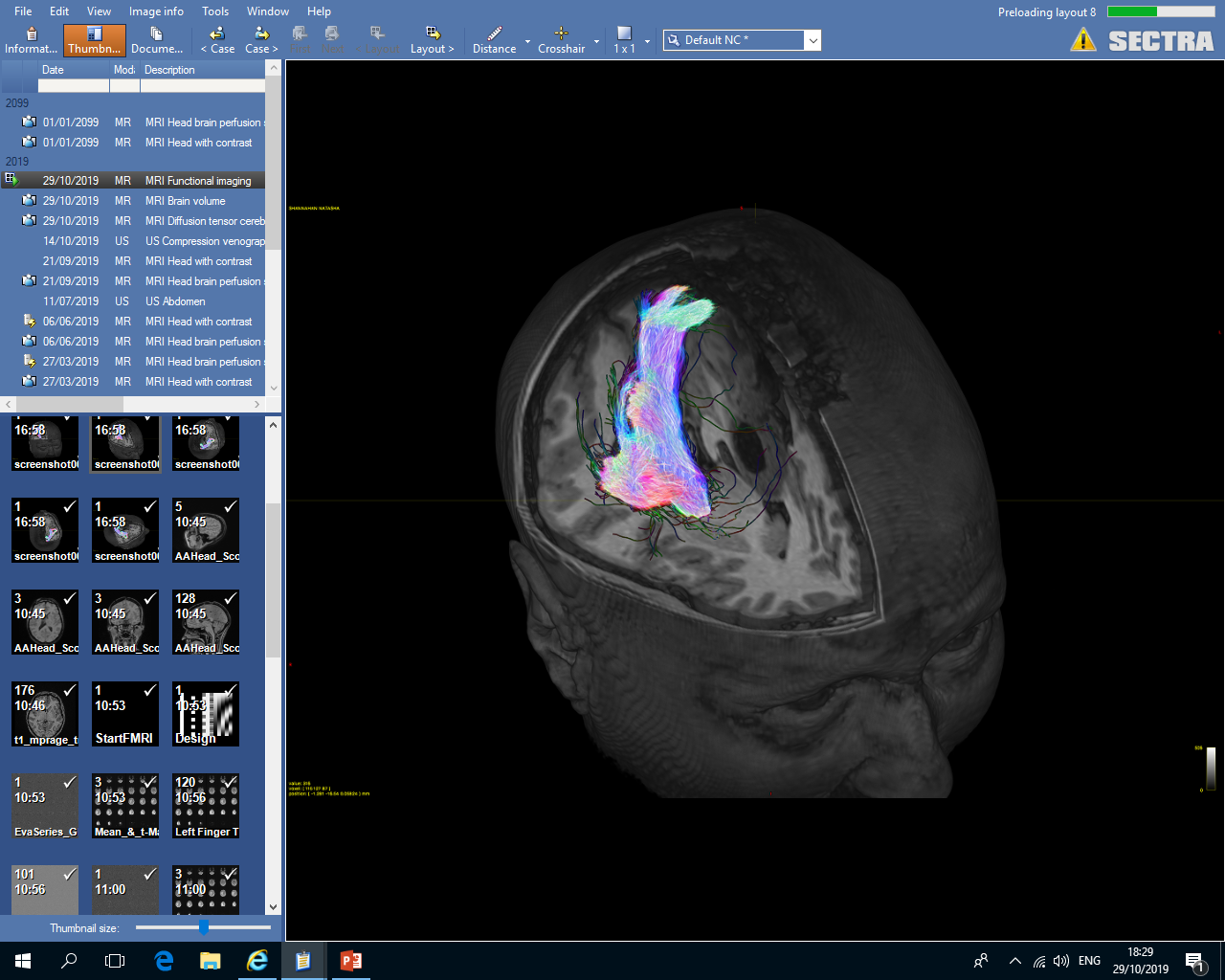
**

c

a

b

d

**Supplementary Figure 2 (a,b,c,d): Pre-operative 3D Reconstruction of Cortical and subcortical corticospinal tract(*foot: blue circle, hand:orange circle, face: yellow circle representation*) utilizing Constrained Probabilistic tractography and modelling (MRTrix opensource software** (Tournier *et al*, 2019)***(****36y left-handed female with recurrent right frontal transformed WHO Grade III IDH 1 mutant MGMT methylated anaplastic gemistocytic astrocytoma who underwent subtotal resection*)

**
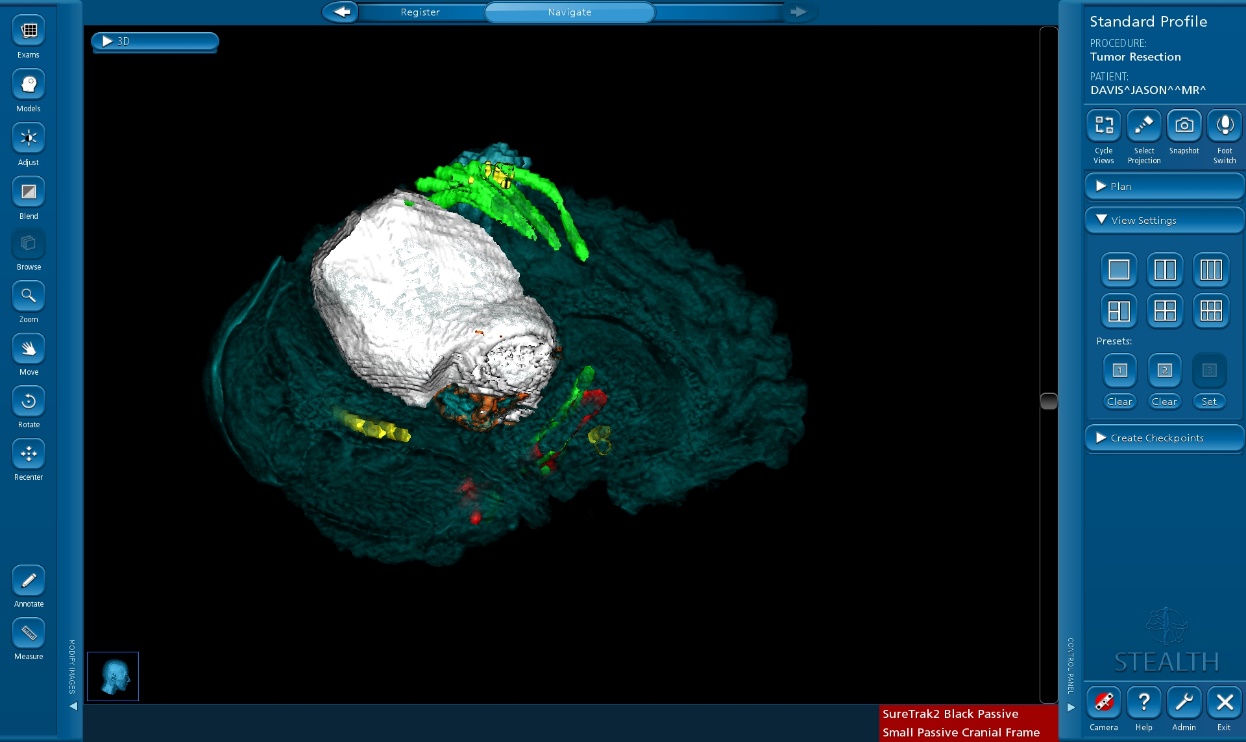

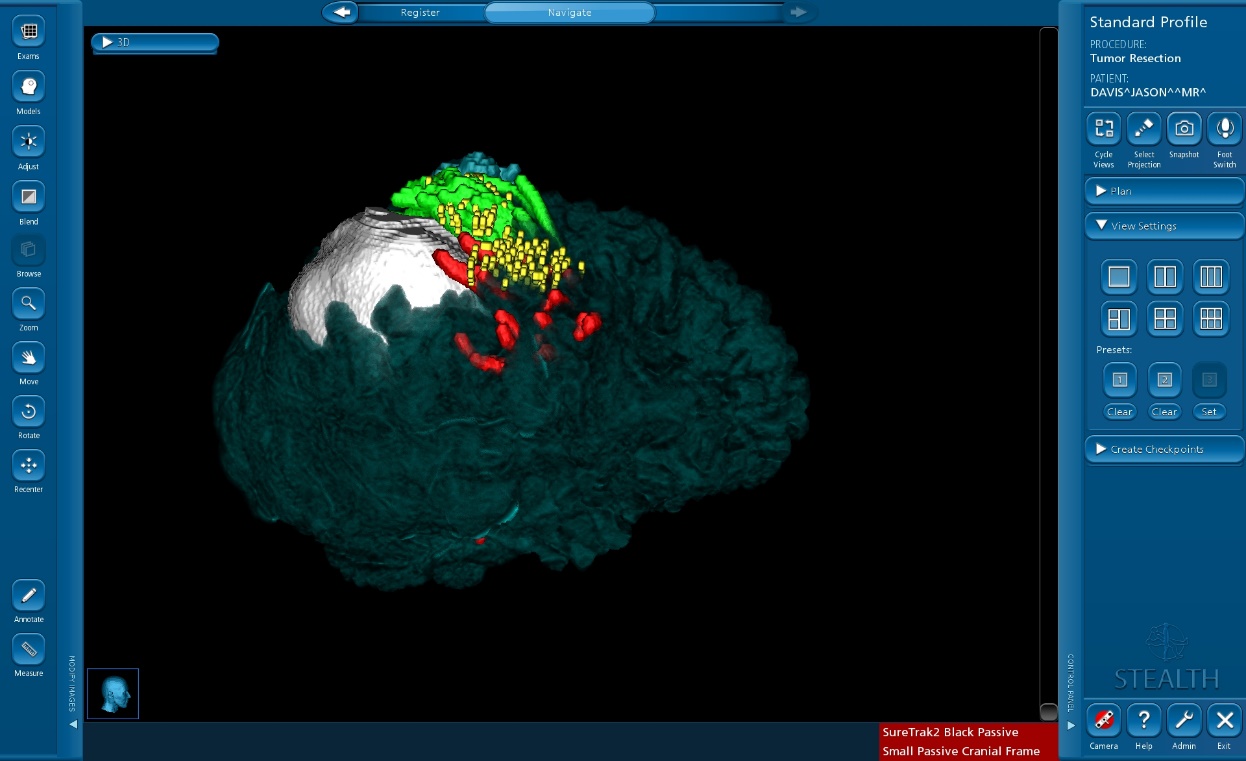

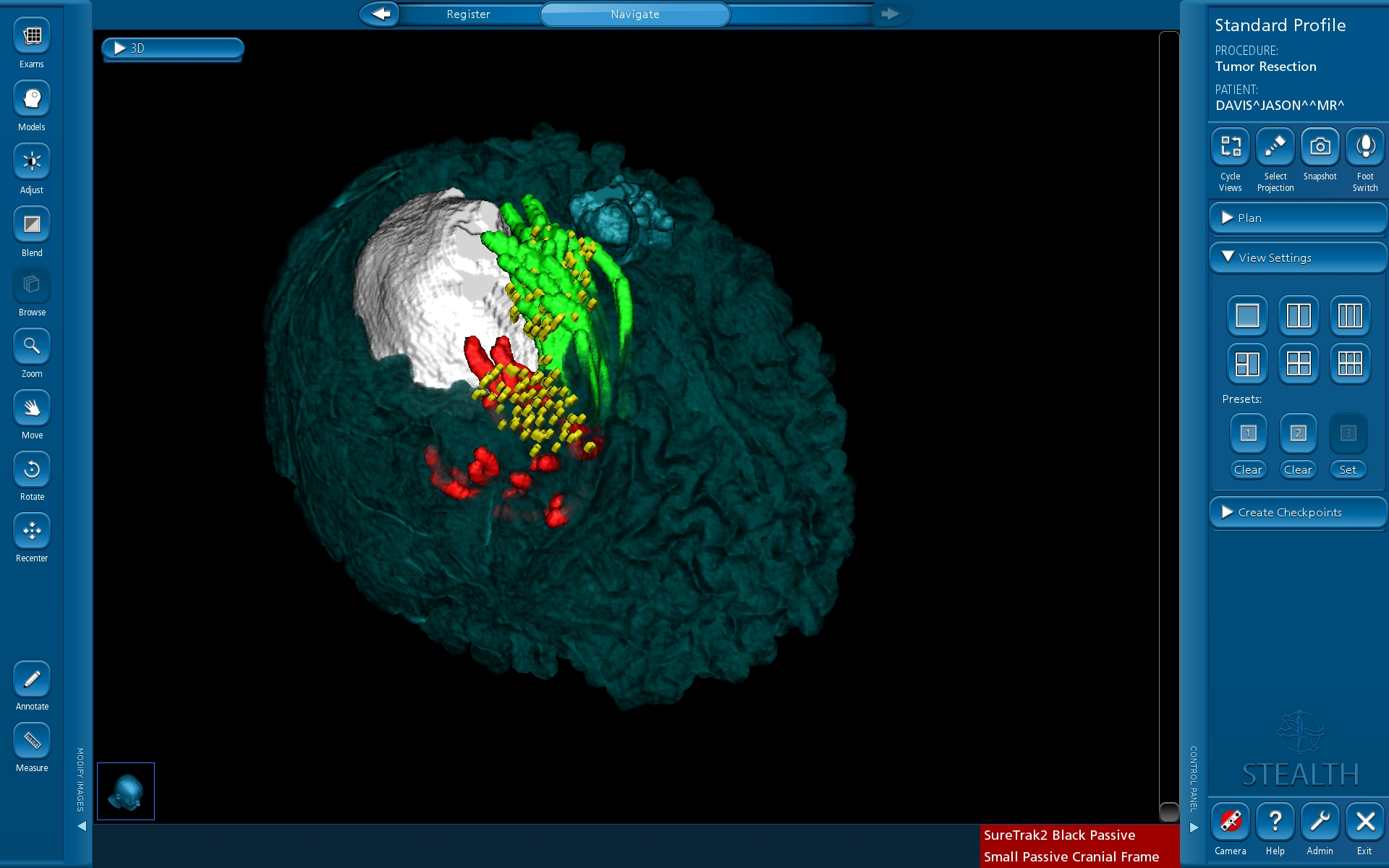
**

c

b

a

**Supplemental Figure 3 (a,b,c): Integration of nTMS motor stimulation points with DTI reconstruction with neuronavigation (StealthStation Medtronic S7 machine, StealthViz software) *(White -Tumour, Red – CST (Face + Upper Limb), Green – CST (Lower Limb), Yellow dots – TMS Motor map, Blue Tractography – CST (contralateral to the lesion****))(22y male with right cystic-solid parietal WHO grade I IDH wildtype, ATRX wildtype, MGMT unmethylated pilocytic astrocytoma who underwent gross total resection)*

**
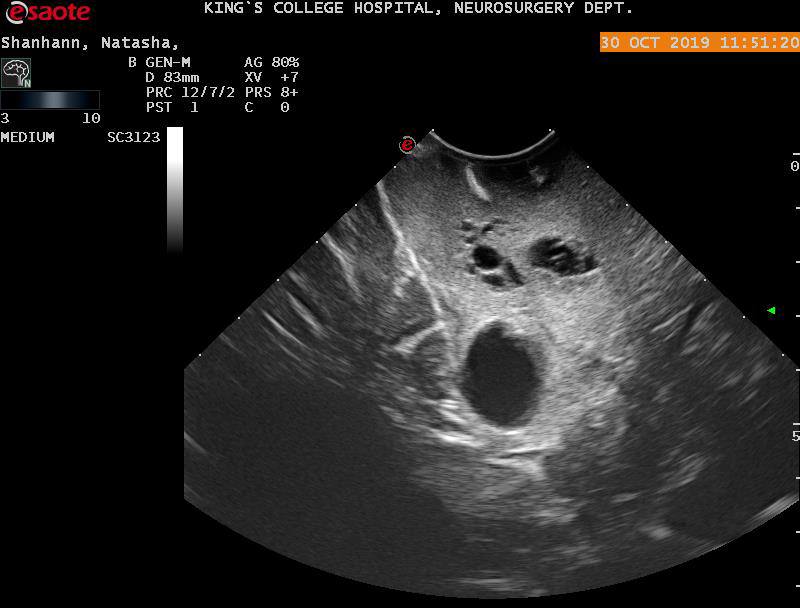

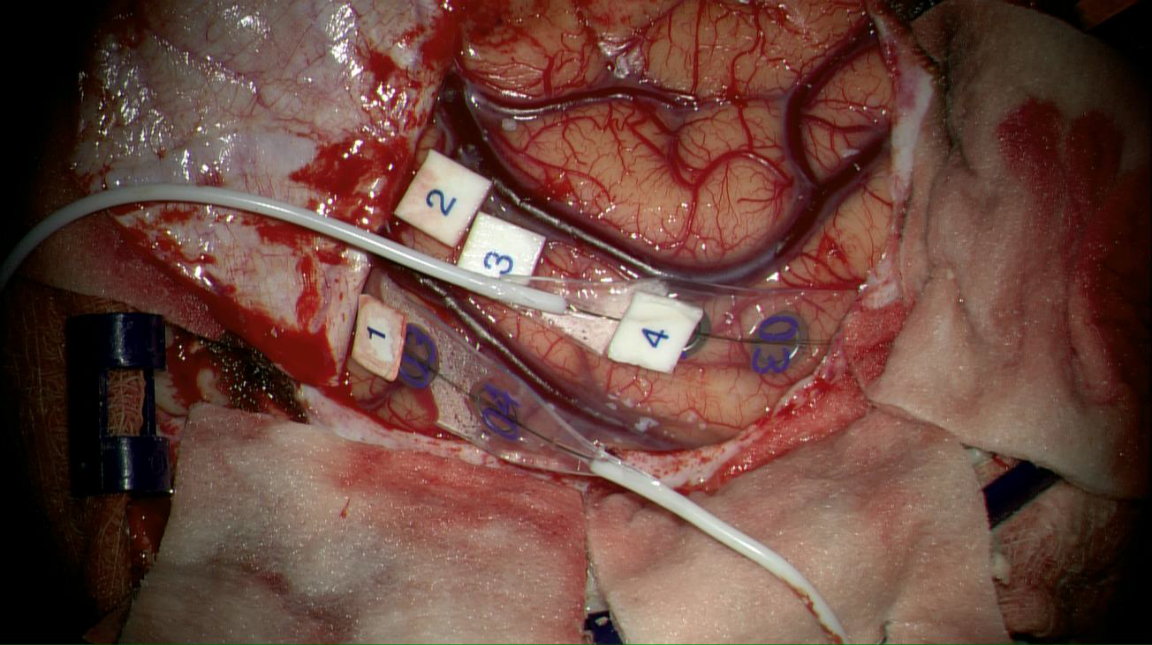

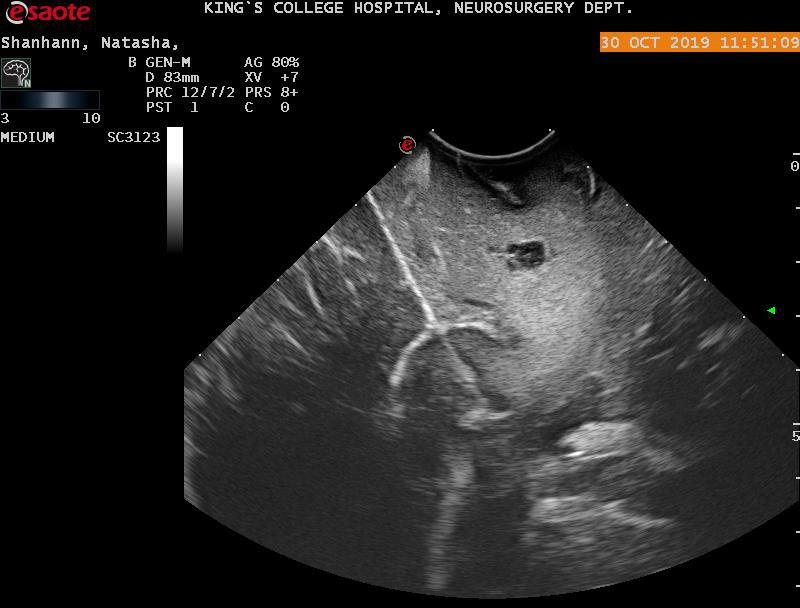

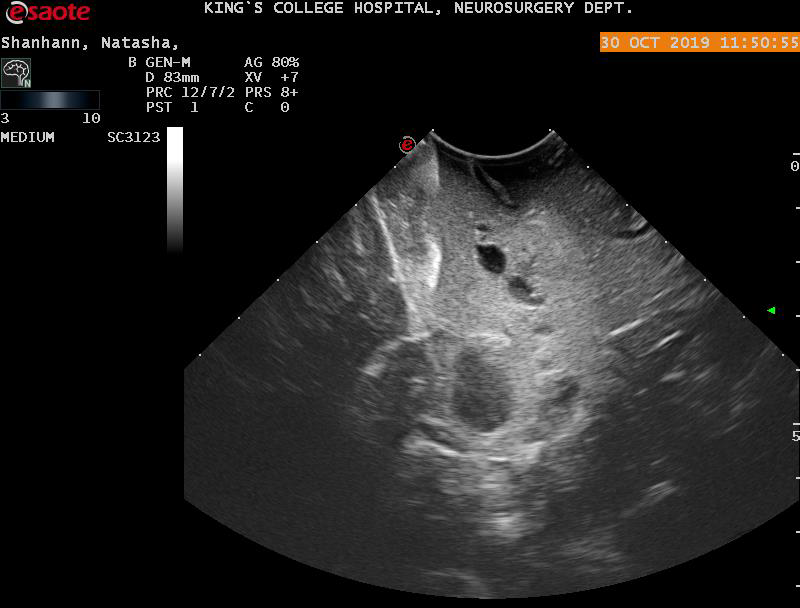
Supplemental Figure 4 (a,b,c) :Intraoperative pre-resection ultrasound (ioUS) demonstrating right frontal tumour and its relation to the pre-central cortex at the cortical and subcortical level ; (d) : Intraoperative image with cortical mapping (**Tag 1 – Lower Limb, Tag 2 – Lower Limb and Intercostal Muscles, Tag 3 – Intercostal Muscles, Tag 4 – Forearm, Strip Number 1 – Monitoring the Foot, Strip Number 2 – Monitoring the Hand)(Black arrow: tumour location, blue arrow: cortical motor cortex and subcortical CST)***(****36y left-handed female with recurrent right frontal transformed WHO Grade III IDH 1 mutant MGMT methylated anaplastic gemistocytic astrocytoma who underwent subtotal resection*)

c

b

a

d

**
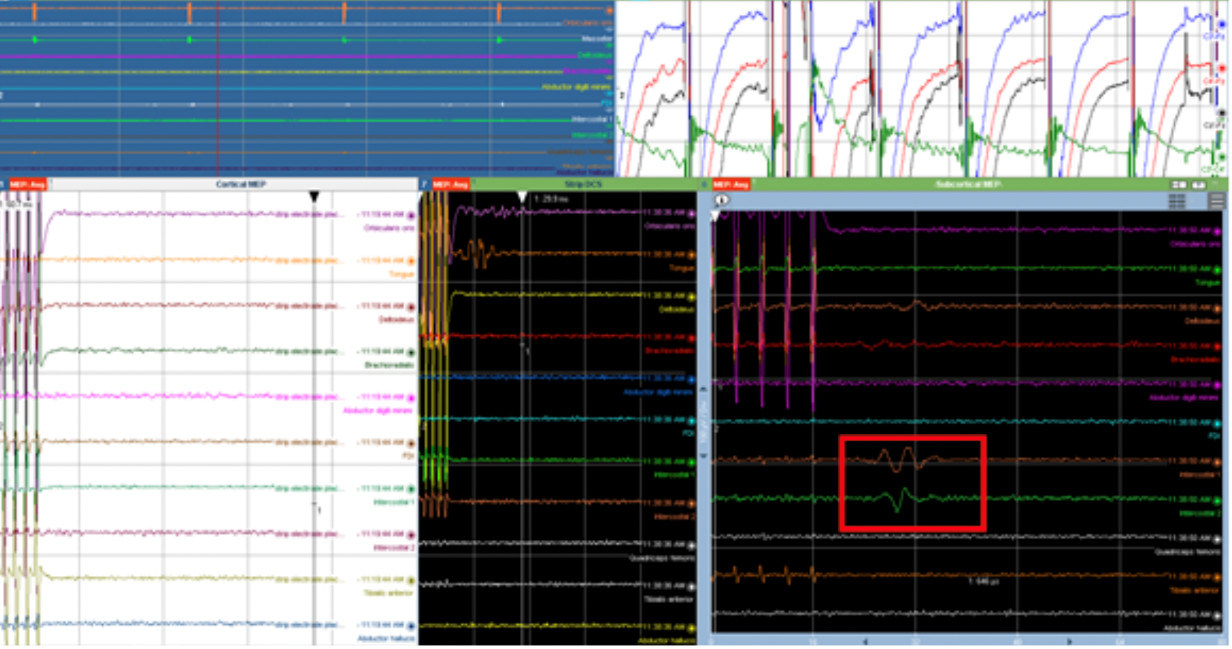
**

**Supplemental Figure 5: Isolated subcortical stimulation of intercostal muscles during intraoperative subcortical mapping**

**
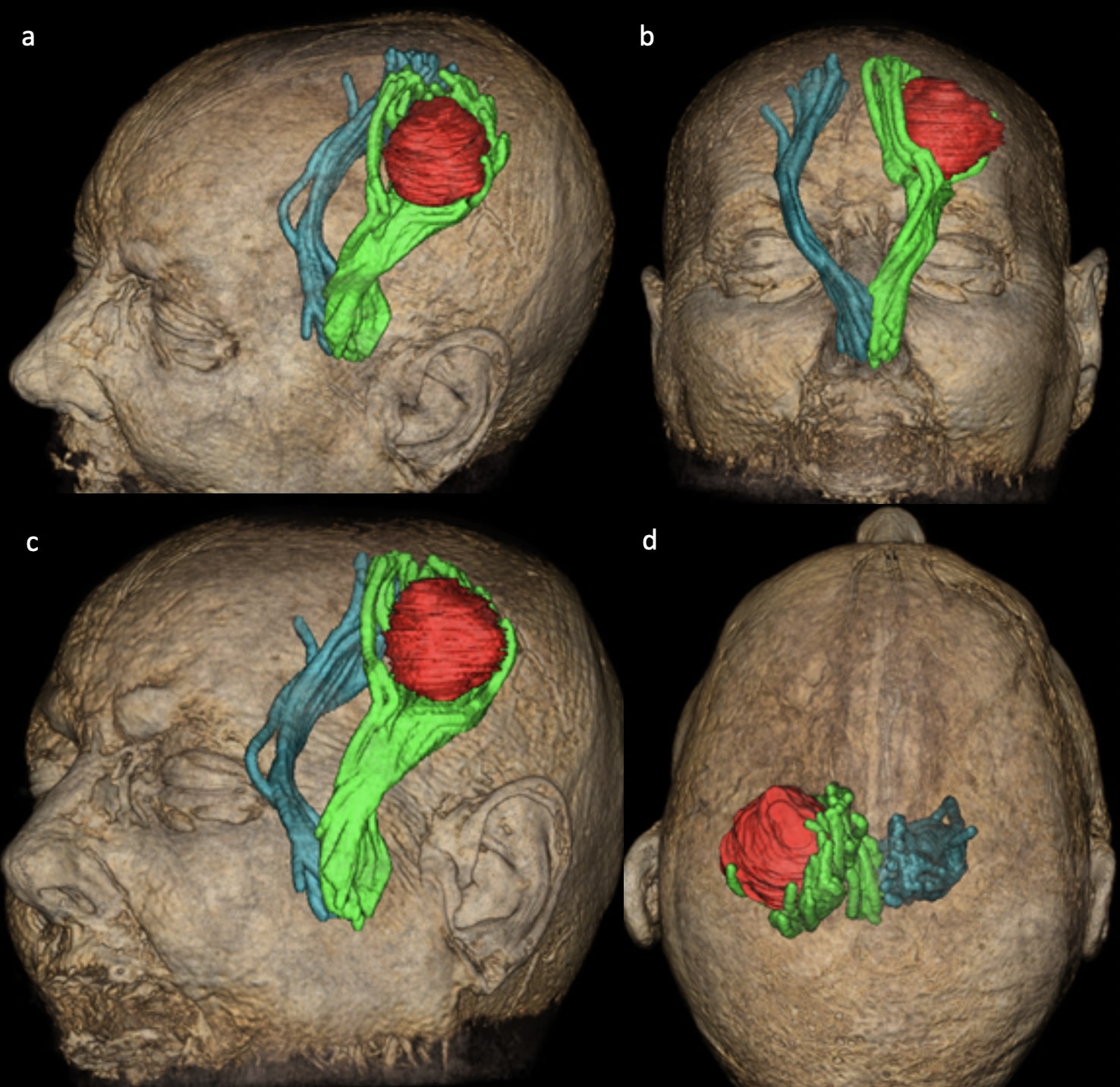
**

**Supplemental Figure 6(a,b,c,d)**: **Pre-operative 3D reconstruction of Corticospinal tract (*blue, green)* with tumour (*red*) utilising the pre-operative DTI and modelling using neuronavigation (Medtronic Stealth S7, StealthViz Software)** *(70y female with left precentral metastatic tumour (primary lung small cell carcinoma who underwent gross total resection)*

**Supplemental Table 1: Literature review of subcortical intraoperative mapping demonstrating each mapped area**

| **SN** | **Articles** | **N** | **Technique** | **Pathology** | **Mapped Areas** | | | | | **MT (mA)** |
| --- | --- | --- | --- | --- | --- | --- | --- | --- | --- | --- |
|  |  |  |  |  | **Leg** | **Trunk** | **Arm** | **Hand** | **Face** |  |
| 1. | (Duffau *et al*, 2003) | 39 | Bipolar | Glioma | + | - | + | + | + | - |
| 2. | (Mikuni *et al*, 2007 ) | 21 | Monopolar  Train of 4 (cortical)  Bipolar train of 4 (subcortical) | Glioma  Cavernoma  Ependymoma | + | - | + | + | - | 5-15 |
| 3. | (Bello *et al*, 2008) | 57 | Monopolar | Glioma | + | - | + | + | - | 3.5-16 |
| 4. | (Vassal *et al*, 2013) | 10 | Monopolar | Glioma | **+** | **-** | **+** | **+** | **+** | **-** |
| 5. | (Bello *et al*, 2014) | 591 | High frequency (monopolar) vs Low frequency (bipolar) | Glioma | + | - | + | + | + | HF: 5-13  LF: 2-6 |
| 6. | (Ghimire *et al*, 2019) | 1 | Monopolar  (Train of 5) | Glioma | - | +  Intercostal muscle | - | - | - | 5 |

*MT: motor threshold, mA: milliamperes*

**Supplemental Table 2: Case series of cortical and subcortical intercostal muscles stimulation (MEP)**

| **SN** | **Age** | **Location of lesion** | **Histopathology** | **Cortical**  **stimulation** | **Subcortical**  **stimulation** | **nTMS pre-op** |
| --- | --- | --- | --- | --- | --- | --- |
| **1** | 53y | Right SMA | Anaplastic Oligodendroglioma Grade III  (IDH -, ATRX preserved) | N | Y; 4mA | Yes |
| **2** | 64y | Right parietal | Metastatic malignant melanoma | N | Y; 4mA | Yes |
| **3** | 35y | Right SMA | Oligodendroglioma Grade III (IDH1 mutant, ATRX preserved) | N | Y; 5mA | Yes |
| **4** | 69y | Left Precentral | Radiation induced Vascular malformation | Y; 14 mA | Y; 6mA | No |
| **5** | 31y | Right parietal | Glioblastoma Grade IV (IDH wild; ATRX preserved; MGMT unmethylated) | N | Y; 6mA | No |
| **6** | 62y | Left Precentral | Metastatic Carcinoma Lung | Y;12mA | N | Yes |
| **7** | 41y | Left Central lobule | Metastatic Breast Carcinoma *P800002* | Y: 10mA | N | Yes |
| **8** | 56y | Left precentral | Metastatic lung Carcinoma *A851813* | Y: 6mA | Y; 12 mA | Yes |
| **9** | 57y | Right frontal | Anaplastic oligodendroglioma (IDH-mutant, grade III IDH1 R132H clone positive (IHC) ATRX retained) *R210577* | Y: 10mA | Y | Yes |
| **10** | 36y | Right frontal | Anaplastic gemistocytic astrocytoma (IDH mutant, WHO grade III ATRX lost) *M027196* | Y | N | Yes |
| **11** | 36y | Left precentral | Glioblastoma, IDH-wildtype, grade IV (WHO)  (IDH wildtype ATRX expression retained Unmethylated MGMT promoter) | N | Y; 5mA | Yes |

*nTMS: navigated transcranial magnetic stimulation; SMA: supplementary motor area; IDH: isocitrate dehydrogenase ;ATRX:* *alpha-thalassemia/mental retardation, X-linked; MGMT:* *Methyl Guanine Methyl Transferase ;IHC: immunohistochemistry ; mA: milliampere*

**Methods:**

**Monopolar cortical and subcortical Stimulation**

Monopolar stimulation is commonly utilised at our institution. It has been shown that the monopolar probe emits a radial, homogenous electrical field different to focused bipolar inter-tip electrical field. We deliver the stimulation with train of 5 monopolar rectangular electrical pulses with inter-stimuli interval of 4.0 ms, pulse width of 0.5ms, at 1Hz and anodal pole for cortical and cathodal pole for subcortical mapping. This high frequency short train stimulation provides a time-locked MEP response with a defined latency and easily quantifiable amplitude. This aids for the motor thresholds to evaluate current-to-distance relationship. These homogenous electrical field and the current-to-distance relation provides an estimate of distance from the corticospinal tract(CST) helping safe maximal resection. This technique has also been proposed to lower the incidence of intra-operative seizures allowing smooth uninterrupted stimulation *(Seidel K et al, 2013; Schucht P et al, 2017; Lavrador et al, 2020)*.

**fMRI Protocol and Analysis**

The MRI protocol included a 3D T1-weighted MPRAGE anatomical sequence (TE/TR = 3.02/2200 ms, voxel = 1 mm^3^), a diffusion spin echo EPI sequence (TE/TR = 86/9500ms, voxel = 2.5mm^3^, b = 0, 1500s/mm^2^, 64 directions), and, for each motor fMRI paradigm, a BOLD contrast gradient echo EPI sequences (TE/TR = 40/3000 ms, voxel = 2.5x2.5x3 mm^3^). The fMRI paradigms consisted of six cycles of alternating rest and activation periods of 30 seconds each, and included finger tapping, foot rocking and lip pouting. The fMRI data was processed employing SPM12 (Wellcome Trust Centre for Neuroimaging, University College London, UK) and in-house developed processing scripts, which included the following:

1) motion correction (rigid body and least square algorithms, SPM12)

2) co-registration to anatomical data (non-linear mutual information algorithm, SPM12)

3) smoothing with an isotropic Gaussian kernel (8 mm full width at half maximum).

Statistical parametric maps (general linear model framework) were calculated using a Student’s t-test with a family-wise error rate significance level set at 0.05. Threshold t-maps were used to define the activation regions. White matter tracts were reconstructed using constrained spherical deconvolution (CSD) algorithms and probabilistic tractography in MRtrix3 (v. 0.3.14, http://www.mrtrix.org/).
